# MMSE is an independent prognostic factor for survival in primary central nervous system lymphoma

**DOI:** 10.1007/s11060-021-03708-8

**Published:** 2021-02-21

**Authors:** Matthijs van der Meulen, Linda Dirven, Katerina Bakunina, Martin J. van den Bent, Samar Issa, Jeanette K. Doorduijn, Jacoline E. C. Bromberg

**Affiliations:** 1 Department of Neuro-Oncology, Erasmus MC Cancer Institute, Brain Tumor Center, University Medical Center Rotterdam, Dr. Molewaterplein 40, 3015 GD Rotterdam, Netherlands; 2 Department of Neurology, Leiden University Medical Center, Leiden, Netherlands; 3 Department of Neurology, Haaglanden Medical Center, The Hague, Netherlands; 4 Department of Hematology, HOVON Data Center, Erasmus MC Cancer Institute, Rotterdam, Netherlands; 5 Department of Hematology, Middlemore Hospital, Auckland, New Zealand; 6 Department of Hematology, Erasmus MC Cancer Institute, University Medical Center Rotterdam, Rotterdam, Netherlands

**Keywords:** MMSE, Primary central nervous system lymphoma, Prognosis

## Abstract

**Introduction:**

To assess the value of the Mini-Mental State Examination (MMSE)-score at baseline in predicting survival in adult primary central nervous system lymphoma (PCNSL) patients.

**Methods:**

In the HOVON 105/ ALLG NHL 24 phase III study patients with newly-diagnosed PCNSL were randomized between high-dose methotrexate-based chemotherapy with or without rituximab. Data on potential (MMSE-score), and known baseline prognostic factors (age, performance status, serum LDH, cerebrospinal fluid total protein, involvement of deep brain structures, multiple cerebral lesions, and the IELSG-score) were collected prospectively. Multivariable stepwise Cox regression analyses were used to assess the prognostic value of all factors on progression-free survival (PFS) and overall survival (OS) among patients with available MMSE score at baseline. Age was analyzed as continuous variable, the MMSE-score both as a continuous and as a categorical variable.

**Results:**

In univariable analysis, age, MMSE-score and whether the patient received rituximab were statistically significantly prognostic factors for PFS. Age and MMSE-score were statistically significantly associated with OS. In a multivariable analysis of the univariately significant factors only MMSE-score was independently associated with the survival endpoints, as a continuous variable (HR for PFS 1.04, 95% CI 1.01–1.08; OS 1.06 (95% CI 1.02–1.10) and as categorical variable HR (< 27 versus ≥ 27 for PFS 1.55 (1.02–2.35); OS 1.68 (1.05–2.70). In our population, performance status, serum LDH, and CSF protein level were not of prognostic value.

**Conclusion:**

Neurocognitive disturbances, measured with the MMSE at baseline, are an unfavorable prognostic factor for both PFS and OS in adult PCNSL patients up to 70 years-old.

**Supplementary Information:**

The online version contains supplementary material available at 10.1007/s11060-021-03708-8.

## Introduction

Primary central nervous system lymphoma (PCNSL) is a rare non-Hodgkin lymphoma confined to the brain, leptomeninges, spinal cord and eyes. Over the last decades prognosis has improved significantly [1]. Although several prognostic factors have been identified and prognostic models have been developed, it remains difficult to predict the prognosis of individual patients.

Two prognostic models are currently widely used in PCNSL patients: the externally validated Memorial Sloan Kettering Cancer Center (MSKCC) prognostic score: [2]age (> 50 years-old) and Karnofsky Performance score (KPS; < 70), and the International Extranodal Lymphoma Study Group (IELSG) score: age (> 60 years-old), WHO/ECOG Performance Status (PS; > 1), Lactate dehydrogenase (LDH) serum level, cerebrospinal fluid (CSF) protein level and involvement of deep brain structures [3].

The Mini-Mental State Examination (MMSE) [4] is a crude screening tool for neurocognitive impairment. In low- and high-grade glioma, the MMSE-score was an independent prognostic factor for both progression free survival (PFS) and overall survival (OS) [5, 6].

In PCNSL patients, data regarding the prognostic value of the MMSE are scarce, despite the fact that cognitive symptoms occur frequently (up to 43%) in this disease [7]. One study describes 95 elderly (> 60 years-old) PCNSL patients, and found that MMSE-score ≤ 24 was the only independent prognostic factor for OS, while age and PS were not [8]. In the present study we aimed to assess whether the MMSE-score at baseline was independently prognostic for both PFS and OS, in a large trial population with adult PCNSL patients up to 70 years-old.

## Methods

### Patients

Patients in the HOVON 105/ALLG NHL 24 study, a large multicenter phase III randomized controlled trial (RCT) in immunocompetent adults with newly diagnosed CD20 positive B-cell PCNSL with WHO/ECOG PS 0–3, were included [9]. The treatment regimen consisted of two cycles of high-dose methotrexate-based chemotherapy, with or without rituximab, followed by high-dose-cytarabine. Patients < 61 years-old subsequently received 30 Gy whole brain radiotherapy. The study was approved by the ethics committee at all participating centers and all participants gave informed consent. Patients underwent an MMSE at baseline, before chemo(-immuno)therapy was initiated, if possible.

### Baseline characteristics

All patients for whom an MMSE-score at baseline was available were included in this study. In addition, the following information was collected: sex, age, WHO/ECOG PS, treatment arm, CSF protein and serum LDH levels at baseline and whether the patient had multiple cerebral lesions, involvement of deep brain structures (periventricular regions, basal ganglia, brainstem and/ or cerebellum), and whether they received rituximab.

### Statistical analysis

First, baseline characteristics, treatment details and survival between those who participated in this side-study and those who could not due to missing MMSE-scores at baseline were compared to assess possible imbalances. Differences were tested using a Chi-Square test for categorical data, and a Kruskal–Wallis test for continuous data. In addition, the median MMSE score per IELSG-score (i.e. 0–1, 2–3, and 4–5) was calculated.

Subsequently, all the above mentioned individual prognostic factors, as well as the composite IELSG-score, were assessed for association with PFS and OS using univariable Cox regression analysis. PFS was defined as time from randomization to progression, relapse or death from any cause, whichever came first. OS was defined as time from randomization to death from any cause, which are the same definitions as used in the HOVON 105/ ALLG NHL 24 trial [9]. Patients still alive at the date of last contact were censored. MMSE was included both as a continuous variable and as categorical variable (< 27 or ≥ 27). The cut-off of 27 was chosen, based on previous recommendations [10, 11]. Age was included as a continuous variable. ECOG PS (≤ 1 versus > 1), serum LDH (above versus below local upper limit of normal), and CSF protein (above versus below cut-off values according to the IELSG score[3]) were included as categorical variables. Factors that were statistically significant in univariable analysis were included in the stepwise multivariable Cox proportional hazards models. A p-value < 0.05 was considered statistically significant. All analyses were performed with Stata version 15.

## Results

MMSE-score at baseline was available for 153 of the 199 (77%) trial patients. There were no significant differences between those who were included and those who were not regarding baseline characteristics and survival, Supplemental Table 1 and Supplemental Fig. 1a and b.

**Table 1:** Univariate and multivariate Cox regression analysis for all risk factors with MMSE as a continuous variable for the progression-free survival and overall survival

	n	Univariate		Multivariate	
		HR (95% CI)	p	HR (95% CI)	p
Progression-free survival					
Female	153	0.85 (0.57–1.28)	0.44		
Age (increase; unit = 10 years)	153	1.33 (1.04–1.71)	0.025	1.28 (0.99–1.65)	0.061
WHO/ECOG > 1	153	0.92 (0.57–1.50)	0.74		
Multiple lesions	138	0.89 (0.58–1.37)	0.59		
Deep structures involved	153	1.39 (0.92–2.09)	0.39		
Elevated CSF total protein	93	0.78 (0.45–1.37)	0.40		
LDH > ULN	153	1.19 (0.77–1.82)	0.44		
Rituximab	153	0.66 (0.44–1.00)	0.049	0.69 (0.45–1.04)	0.075
MMSE (decrease unit = 1 point)	153	1.05 (1.01–1.08)	0.0042	1.04 (1.01–1.08)	0.008
IELSG-score	153	0.74 (0.48–1.16)	0.20		
Overall survival					
Female	153	1.12 (0.71–1.76)	0.64		
Age (increase; unit = 10 years)	153	1.36 (1.02–1.82)	0.036	1.32 (0.97–1.77)	0.069
WHO/ECOG > 1	153	1.29 (0.77–2.16)	0.32		
Multiple lesions	138	1.01 (0.62–1.63)	0.97		
Deep structures involved	153	1.25 (0.79–1.99)	0.34		
Elevated CSF total protein	93	0.64 (0.33–1.26)	0.20		
LDH > ULN	153	1.15 (0.71–1.88)	0.57		
Rituximab	153	0.86 (0.55–1.35)	0.51		
MMSE decrease unit = 1 point)	153	1.06 (1.02–1.10)	0.001	1.06 (1.02–1.10)	0.002
IELSG-score	153	0.66 (0.40–1.10)	0.11		

Hazard ratio’s (HR) and 95% confidence intervals (CI) are shown with their p-value. *WHO* World Health Organization, *ECOG* Eastern Cooperative Oncology Group, *CSF* cerebrospinal fluid, *LDH* lactate dehydrogenase, *ULN* upper limit of normal, *MMSE* mini-mental state examination, *IELSG* International Extranodal Lymphoma Study Lymphoma Study Group

**Fig. 1:**
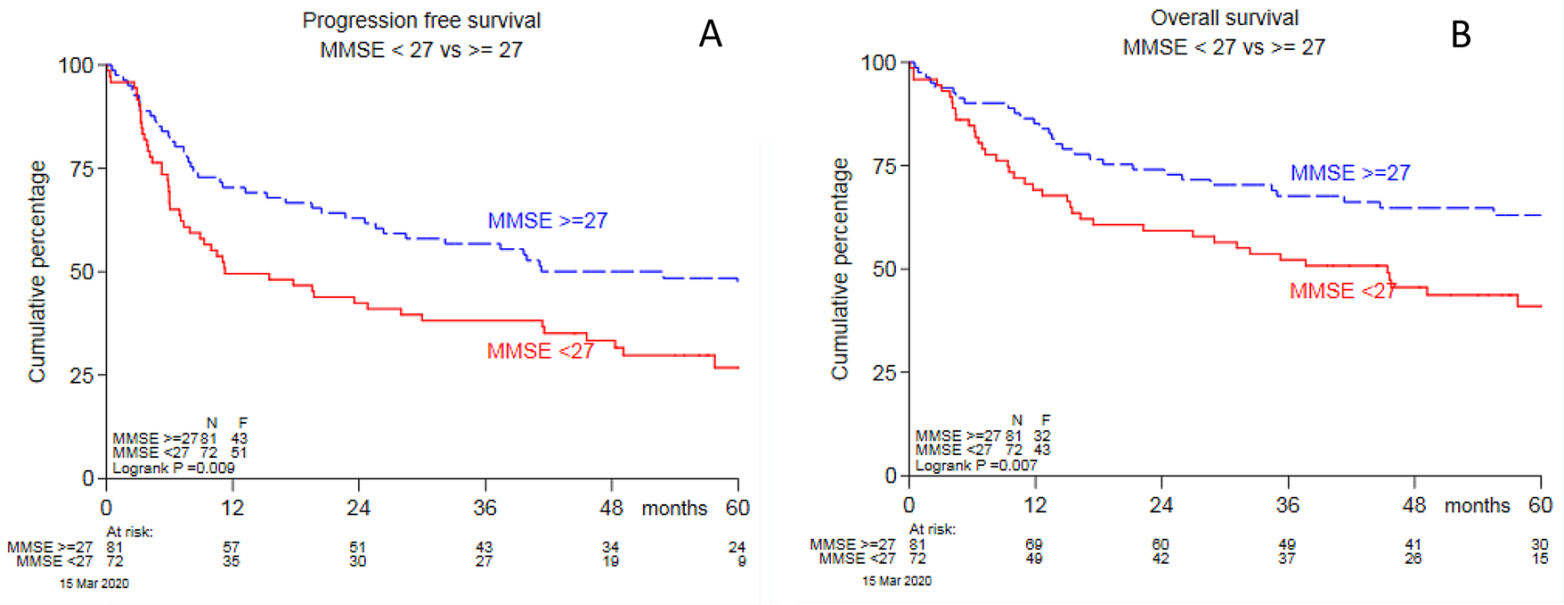
**a** Progression free survival and **b** overall survival for those with an MMSE-score of < 27 and ≥ 27 at baseline

Stratified by the IELSG-score, there was difference in median (range) MMSE-score at baseline: 29 (11–30) in the IELSG 0–1 group and 25 (6–30) in the 2–3 group. In the IELSG 4–5 group the median score was 26 (7–29), but only 5 patients were in this group, Supplemental Table 2).

**Table 2:** Multivariate Cox regression analysis for univariately significant risk factors with MMSE as a categorical variable for the progression-free survival and overall survival

	Multivariate	
	HR (95% CI)	p
Progression-free survival		
Age (increase; unit = 10 years)	1.24 (0.95–1.60)	0.109
Rituximab	0.70 (0.47–1.05)	0.087
MMSE < 27	1.55 (1.02–2.35)	0.040
Overall survival		
Age (increase; unit = 10 years)	1.26 (0.94–1.71)	0.127
MMSE < 27	1.68 (1.05–2.70)	0.031

Hazard ratio’s (HR) and 95% confidence intervals (CI) are shown with their p-value. *MMSE* mini-mental state examination

In the univariable regression analyses age, receipt of rituximab and baseline MMSE-score were associated with PFS. Only age and MMSE were statistically significant predictors of OS (Table 1). In multivariable analysis, only MMSE-score at baseline was independently associated with both PFS and OS. We found that each unit decrease in MMSE-score was associated with a poorer prognosis: for PFS (Hazard Ratio [HR], 95% confidence interval [CI] 1.04, 1.01–1.08) and OS (HR 95% CI 1.06, 1.02–1.10), Table 2. When including the MMSE-score as categorical variable in multivariable analyses, corrected for age and rituximab, a baseline-score < 27 (as compared to a score ≥ 27) was the only factor associated with PFS (HR 1.55, 95% CI 1.02–2.35) and overall survival (HR 1.68, 95% CI 1.05–2.70), Table 2 and Fig. 1. After adding the IELSG-score to the other prognostic factors in the multivariable analysis, the MMSE-score at baseline remained the only independent prognostic factor for both PFS and OS (Table 1).

## Discussion

In this large, prospectively examined study-population of PCNSL patients, we found that the MMSE-score at baseline, both as a continuous variable and as a categorical variable (< 27), is an independent prognostic factor for both PFS and OS. MMSE was not evaluated in either of the two most-used prognostic scores in PCNSL but our data suggest this factor is the most valuable for predicting outcome [2, 3].

Our results are consistent with a previously published analysis performed in elderly PCNSL patients: those with an MMSE-score ≤ 24 had a worse OS than those with a score > 24 [8]. Moreover, in a recent RCT among patients up to 60 years the Mattis Dementia Rating Scale, another screening tool for neurocognitive impairment, was significantly associated with OS, though in univariable analysis only. In multivariable analysis, only WHO/ECOG PS was associated with both PFS and OS [12]. In this study we chose a score of 27 as cut-off of to distinguish patients with normal and impaired cognition. This cut-off was based on population-based norms [13], and is supported by the finding that the median MMSE score in our population also was 27. Moreover, in low-grade glioma an MMSE score of 29 has been found to be predictive for survival [14], compared to a score of 27 in our population. This difference is likely explained by the more diffuse nature of PCNSL, like Alzheimer’s disease, in which a cut-off score of 27 was found to be more sensitive to detect cognitive dysfunction [15].

Age and PS are common prognostic factors in oncology patients. In our study, both factors were not independently prognostic for survival in multivariable analysis, although age showed a trend towards significance both for PFS (p = 0.061) and OS (p = 0.069). For age, this might be explained by the small number of patients ≤ 50 years-old and the exclusion of patients > 70-years-old in this study. Some other studies also did not find a prognostic effect of age, even as categorical variable, although these studies included only younger or only elderly patients [8, 12, 16]. Categorizing age has been very useful for stratifying patients in clinical trials, but ageing is a continuous process. So, from a biological perspective, it is more logical to include age as a continuous variable. Similarly, in contrast to most other studies [2, 3, 12] we did not find an effect of the WHO/ECOG PS on survival. A relatively low power of the test might explain this finding. In our study population, only 25% had an ECOG > 1, which was the cut-off for the ECOG/ WHO performance score that was used in the IELSG-model. This small proportion of patients with an ECOG > 1 (patients with a WHO/ECOG PS of 4 were ineligible for the HOVON 105/ALLG NHL 24 study) may have influenced the power of the analysis. Although some other studies [8, 16] also did not identify a prognostic effect of performance status, it remains unexpected.

Besides age, none of the other factors, including the IELSG-score showed any relation with survival in our population. However, if we compare the MMSE score in the three IELSG categories we see a clear difference. This suggests that a higher IELSG score at baseline is associated with a lower MMSE-score. But, after including all factors in one prognostic model, only MMSE remains as prognostic factor.

The MMSE was originally developed as a screening tool for cognitive impairment in dementia, and lacks sensitivity in detecting neurocognitive disturbances, particularly in changes over time [17]. A comprehensive neurocognitive assessment with standardized tests is more predictive of survival than brief screening tools [18]. However, the strength of the MMSE is that is available in many languages, the time to completion is limited, and it can be performed by any healthcare worker without extensive training. Therefore, the use of the MMSE may be valuable in clinical practice. In line with the previously mentioned population-based norm (< 27), we would advocate to use the categorized variable in prognostic assessment, because of the clinical relevance and ease of interpretation.

The major strength of our study is the prospective data collection within a large clinical trial resulting in MMSE-scores for the majority of patients and a uniform treatment and evaluation protocol. A limitation is the relatively small number of patients for prognostication; our sample size is smaller than that in the MSKCC (n = 238) and IELSG models (n = 378). A down-side of all studies based on trial patients is that findings may not be generalizable to the whole PCNSL population. Given the relatively low percentage of patients with ECOG > 1 in this substudy and the suggestion of longer survival—though not significant—compared with patients not included (see supplemental data), inadvertent bias in the selection of patients cannot be excluded. This may be the result of more patients in a poor performance status being unable or unwilling to undergo the MMSE. However, the magnitude of the effect is more likely to be underestimated than overestimated with this population of relatively good performance patients. Lastly, we could only include a limited number of potential prognostic factors in our analysis for statistical reasons. The final choice was based on known and/or previously reported relevant factors, and the availability of factors (e.g. specific information on comorbidities was not available). As a result, not all potentially relevant factors were included in the analyses and this may have overestimated the association between the MMSE score and survival. Additionally, WBRT was not included as a factor in the multivariable analyses, although a benefit on PFS has been described [19]. Because WBRT was only part of the treatment in patients under the age of 60, these factors were highly correlated. Since we were limited in the number of factors that could be included in the multivariable models due to limited statistical power, only age was included. As a result of this confounding, the effect of age may be overestimated.

To conclude, the MMSE is an easily assessable and relevant clinical factor which has not been included in prior prognostic studies in patients with PCNSL. In this dataset the MMSE-score at baseline is an independent clinical prognostic factor in adult PCNSL patients up to 70-years-old. If validated in another large population, patients should be counseled with this effect in mind, and other prognostic scores should be re-evaluated.

## Electronic Supplementary Material

Below is the link to the electronic supplementary material.

Supplementary Material 1 (DOCX 65 kb)
