# Supplementary material for: MMSE is an independent prognostic factor for survival in primary central nervous system lymphoma
Source: J Neurooncol. 2021 Feb 21;152(2):357–62. doi: 10.1007/s11060-021-03708-8 (PMC7997829; doi:10.1007/s11060-021-03708-8)
Supplement: Supplementary file 1 — Supplementary Material 1 (DOCX 65 kb) [file 11060_2021_3708_MOESM1_ESM.docx]

**MMSE is an independent prognostic factor for survival in primary central nervous system lymphoma**

Matthijs van der Meulen MD^1^, Linda Dirven PhD^2,3^, Katerina Bakunina MSc^4^, Martin J. van den Bent MD, PhD^1^, Samar Issa MD^5^, Jeanette K. Doorduijn MD, PhD^6^, Jacoline E.C. Bromberg MD, PhD^1^

^1^ Erasmus MC Cancer Institute, Brain Tumor Center, University Medical Center Rotterdam, Department of Neuro-Oncology;

^2^ Leiden University Medical Center, Department of Neurology;

^3^ Haaglanden Medical Center, The Hague, Department of Neurology;

^4^ HOVON Data Center, Department of Hematology, Erasmus MC Cancer Institute, Rotterdam, Netherlands;

^5^ Department of Hematology, Middlemore Hospital, Auckland, New Zealand;

^6^ Erasmus MC Cancer Institute, University Medical Center Rotterdam, Department of Hematology

**Supplemental**

|  | **Patients with baseline MMSE**  n=153 | **Patients without baseline MMSE** n=46 | **Total**  n=199 |
| --- | --- | --- | --- |
| Sex (n, % male) | 83 (54%) | 26 (57%) | 109 (55%) |
| Age (median, IQR) | 61 (55-67) | 61 (57-66) | 61 (55-67) |
| WHO/ECOG performance score (n, %)  WHO/ECOG 0  WHO/ECOG 1  WHO/ECOG 2  WHO/ECOG 3 | 37 (24%)  78 (51%)  24 (16%)  14 (9%) | 6 (13%)  23 (50%)  10 (22%)  7 (15%) | 43 (22%)  101 (51%)  34 (17%)  21 (10%) |
| Comorbidities active at baseline (n, % ≥2) | 76 (50%) | 28 (61%) | 104 (52%) |
| MMSE-score at baseline (median, IQR)  range | 27 (23-29)  6-30 |  |  |
| Multiple lesions  Missing/ NA | 63 (41%)  15 (10%) | 14 (30%)  4 (9%) | 77 (39%)  19 (10%) |
| Deep structures involved (n, %) | 98 (64%) | 27 (59%) | 125 (63%) |
| Serum LDH at baseline (n, % >ULN)  Missing (n, %) | 49 (32%)  - | 9 (20%)  1 (2%) | 58 (29%)  1 (1%) |
| CSF protein level at baseline  (median, range)  Missing (n, %) | 0.70 g/L  (0.03-3.90)  60 (39%) | 0.60 g/L  (0.10-2.10)  23 (50%) | 0.60 g/L  (0.03-3.90)  83 (42%) |
| Dexamethason used (n,%)  Daily dose (mg); median, IQR  Daily dose (mg); range | 127 (83%)  8.0 (4.0-16.0)  1.0-40.0 | 32 (70%)  8.0 (4.0-12.0)  2.0-48.0 | 159 (80%)  8.0 (4.0-12.0)  1.0-48.0 |
| Treatment-arm (n, %)  MBVP  R-MBVP | 76 (50%)  77 (50%) | 24 (52%)  22 (48%) | 100 (50%)  99 (50%) |
| **Study drug exposure** | | | |
| HD-cytarabine (Ara-C) (n, %) | 126 (82%) | 35 (76%) | 161 (81%) |
| WBRT (n, %) | 54 (35%) | 16 (35%) | 70 (35%) |
| Radiation boost given (n, %) | 26 (17%) | 13 (28%) | 39 (20%) |
| Intrathecal treatment given (n, %) | 10 (7%) | 6 (13%) | 16 (8%) |
| **Survival in months** | | | |
| Progression free survival (median, 95% CI) | 28.5 (17.0-48.3) | 15.8 (10.5-39.2) | 26.3 (15.5-41.1) |
| Overall survival (median, 95% CI) | 65.2 (44.7-NR) | 56.6 (27.0-NR) | 61.1 (44.7-75.5) |

**Supplemental Table 1. Baseline clinical and sociodemographic characteristics of the patients who were included and excluded in this study.** IQR = interquartile range, WHO = World Health Organization, ECOG = Eastern Cooperative Oncology Group, NA= not applicable, no brain lesions, LDH = lactate dehydrogenase, CSF = cerebrospinal fluid, (R-)MBVP = (rituximab), methotrexate, tenoposide, BCNU and prednisolone, WBRT = whole-brain radiotherapy, 95% CI = 95% confidence interval, NR= not reached.

| *IELSG score* | *0-1* | *2-3* | *4-5* | *Total* |
| --- | --- | --- | --- | --- |
| *Median MMSE-score (range)* | *29 (11-30)* | *25 (6-30)* | *26 (7-29)* | *27 (6-30)* |

**Supplemental Table 2. Median MMSE score (range) per International Extranodal Lymphoma Study Group (IELSG)-score.** Kruskal-Wallis test p=0.001)


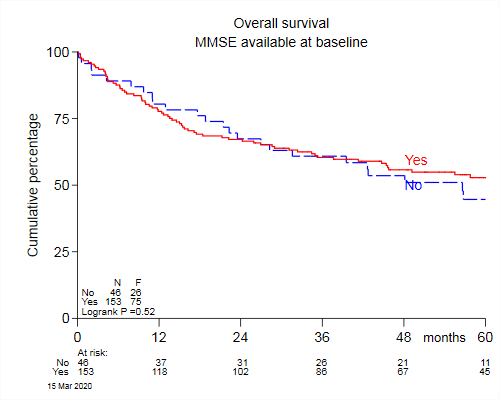

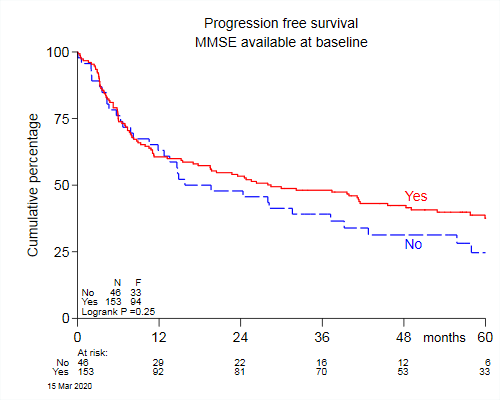


B

A

**Supplemental Figure 1. A. Progression free survival and B. Overall survival for those with an MMSE-score at baseline (included patients in this sub-study) and those without an MMSE-score at baseline.**
